# Transmitted Drug Resistance in Persons with Acute/Early HIV-1 in San Francisco, 2002-2009

**DOI:** 10.1371/journal.pone.0015510

**Published:** 2010-12-10

**Authors:** Vivek Jain, Teri Liegler, Eric Vittinghoff, Wendy Hartogensis, Peter Bacchetti, Lauren Poole, Lisa Loeb, Christopher D. Pilcher, Robert M. Grant, Steven G. Deeks, Frederick M. Hecht

**Affiliations:** 1 HIV/AIDS Division, San Francisco General Hospital, University of California San Francisco, San Francisco, California, United States of America; 2 Department of Epidemiology and Biostatistics, University of California San Francisco, San Francisco, California, United States of America; 3 Gladstone Institute for Virology, University of California San Francisco, San Francisco, California, United States of America; The University of Hong Kong, Hong Kong

## Abstract

**Background:**

Transmitted HIV-1 drug resistance (TDR) is an ongoing public health problem, representing 10–20% of new HIV infections in many geographic areas. TDR usually arises from two main sources: individuals on antiretroviral therapy (ART) who are failing to achieve virologic suppression, and individuals who acquired TDR and transmit it while still ART-naïve. TDR rates can be impacted when novel antiretroviral medications are introduced that allow for greater virologic suppression of source patients. Although several new HIV medications were introduced starting in late 2007, including raltegravir, maraviroc, and etravirine, it is not known whether the prevalence of TDR was subsequently affected in 2008–2009.

**Methodology/Principal Findings:**

We performed population sequence genotyping on individuals who were diagnosed with acute or early HIV (<6 months duration) and who enrolled in the Options Project, a prospective cohort, between 2002 and 2009. We used logistic regression to compare the odds of acquiring drug-resistant HIV before versus after the arrival of new ART (2005–2007 vs. 2008–2009). From 2003–2007, TDR rose from 7% to 24%. Prevalence of TDR was then 15% in 2008 and in 2009. While the odds of acquiring TDR were lower in 2008–2009 compared to 2005–2007, this was not statistically significant (odds ratio 0.65, 95% CI 0.31–1.38; p = 0.27).

**Conclusions:**

Our study suggests that transmitted drug resistance rose from 2003–2007, but this upward trend did not continue in 2008 and 2009. Nevertheless, the TDR prevalence in 2008–2009 remained substantial, emphasizing that improved management strategies for drug-resistant HIV are needed if TDR is to be further reduced. Continued surveillance for TDR will be important in understanding the full impact of new antiretroviral medications.

## Introduction

Transmitted drug-resistant (TDR) HIV is an ongoing public health challenge, affecting approximately 7–21% of new HIV infections in the United States and Europe [1]–[6]. Understanding current TDR patterns can help clinicians assess the importance of genotyping antiretroviral therapy (ART)-naïve patients, inform the selection of ART regimens, and anticipate trends that may affect our future ability to effectively treat the HIV epidemic with existing ART agents.

There are two important sources of TDR: (1) persons who develop drug resistance mutations while on ART and subsequently transmit HIV and (2) persons who acquire TDR mutations during initial infection and maintain the mutations in the absence of ART until they transmit HIV. If TDR trends are driven primarily by persons with drug-resistant HIV who are viremic despite taking ART, changes in ART that achieve better suppression of drug-resistant HIV should rapidly decrease TDR rates. In contrast, if TDR is driven more by ART-naïve individuals, the effects of novel therapies should be minimal or delayed, at least during the initial period that these drugs become widely available.

Treatment options for patients with drug-resistant HIV changed dramatically from 2007–2008. Boosted darunavir and etravirine both showed strong efficacy in clinical trials [7]–[10], and two medications representing novel ART classes—raltegravir and maraviroc [11], [12]—became widely available in the United States and particularly in San Francisco, aided by universal ART access programs.

Although the full clinical impact of these new drugs remains undefined, emerging data suggest that the remarkable efficacy displayed in clinical trials—particularly with raltegravir [13]—is also being observed in routine care [14]. Consistent with these reports, investigators in San Francisco recently reported that from 2004–2008, the city-wide virologic suppression rate improved from 48% to 78% [15]. These investigators also developed a novel population-level HIV risk measure termed “community viral load”—defined as the mean of all persons' most recent HIV plasma RNA levels—and reported that community viral load declined substantially from 2004–2008 period [15].

A recent mathematical modeling study of TDR in San Francisco, however, projected that NNRTI drug resistance would increase over the next five years, primarily from transmission by ART-naïve individuals [16]. Thus far, it has not been possible to compare predictions from this modeling study to actual patient-based observational data from 2008 and 2009.

The effect of the newest ART medications on the transmission of drug-resistant HIV is not known. We therefore analyzed HIV genotypes among patients with acute/early HIV in San Francisco, with a primary objective of estimating the prevalence of TDR in an urban setting with historically high levels of drug resistance [17]. Our secondary objective was to compare the prevalence of TDR before and after the introduction of new antiretroviral agents in late 2007.

## Methods

### Ethics statement

The Committee on Human Research (CHR) is the institutional review board for the University of California, San Francisco and its affiliates, FWA00000068. The CHR approved this study, which involves human subjects as research subjects, entitled “The Options Project: An Observational Study of Individuals Recently Infected with HIV-1.” This study was given approval number H7429-11471-16, which expires 07 May 2011. All participants gave informed written consent for participation.

### Study population and setting

We studied enrollees in the Options Project (San Francisco General Hospital, University of California, San Francisco) with estimated HIV infection dates from 2002–2009. The Options Project is a cohort study of individuals enrolled within 12 months of HIV antibody seroconversion (in 2003, this was restricted to within 6 months of seroconversion). Most participants are referred by community providers if acute (<1 month) or early (<6 months) HIV infection is suspected. Remaining participants directly seek screening, or are referred from community-based organizations and HIV testing sites. Participants are enrolled if they meet screening criteria for acute/early HIV that combine clinical history, serologic testing, and plasma HIV RNA determination as described previously [18], [19]. Briefly, participants were defined as having acute/early HIV if they met one or more of the following three criteria: (1) two plasma HIV-1 RNA levels ≥3,000 copies/mm^3^ with a negative or indeterminate HIV-1 antibody test; (2) a positive HIV-1 antibody test, with a history of a negative HIV-1 antibody test within the previous 12 months (in 2003, this was changed to 6 months); or (3) a clinical history suggestive of recent HIV-1 acquisition, along with a reactive standard HIV-1 antibody test, but a nonreactive less-sensitive (“de-tuned”) HIV-1 antibody test [20], [21].

### Clinical and laboratory evaluations

Study participants had demographic and behavioral data collected via standardized interviews by trained counselors. CD4 cell counts and plasma HIV-1 RNA levels were also measured.

HIV-1 population sequence genotypes were determined on all participants (TRUGENE system, Siemens Healthcare Diagnostics, Tarrytown, NY) as described previously [22], [23]. Initial genotypes were included for analysis if performed within 90 days of screening for cohort entry. For individuals who initiated ART during early HIV infection, genotypes were analyzed only if done within 10 days of initiating ART. Drug resistance was ascertained using published guidelines [24]. These guidelines optimize the specificity of TDR classification for epidemiologic studies by including only mutations that are rarely selected for without drug pressure, and by excluding common polymorphic mutations.

### Statistical methods

The overall prevalence of TDR was calculated by estimated year of infection, along with exact binomial 95% confidence intervals. Resistance to each ART class (nucleoside reverse transcriptase inhibitors [NRTIs], NNRTIs, and PIs) was also calculated by year.

To compare the odds of TDR before and after new antiretroviral medications arrived in late 2007, we divided patients into two groups by estimated dates of HIV infection (2005–2007 vs. 2008–2009). Logistic regression of TDR was performed on this binary time period predictor. Several other predictors of TDR were also examined in unadjusted analyses, including age, injection drug use <2 months prior to cohort enrollment, sexual identity (MSM, male non-MSM, or female), and estimated duration of HIV infection upon specimen collection.

## Results

A total of 372 patients enrolled in the Options cohort from 2002–2009 and had baseline genotyping. Overall, 95% were male, with a median age of 35 years (IQR 30–40 years); 96% were men who have sex with men (MSM), and 9% used injection drugs. The median CD4 cell count at diagnosis was 520 cells/mm^3^ (IQR 391-660 cells/mm^3^); median plasma HIV RNA level was 59,854 copies/mm^3^ (IQR 9,775–404,885 copies/mm^3^).

**Figure 1:**
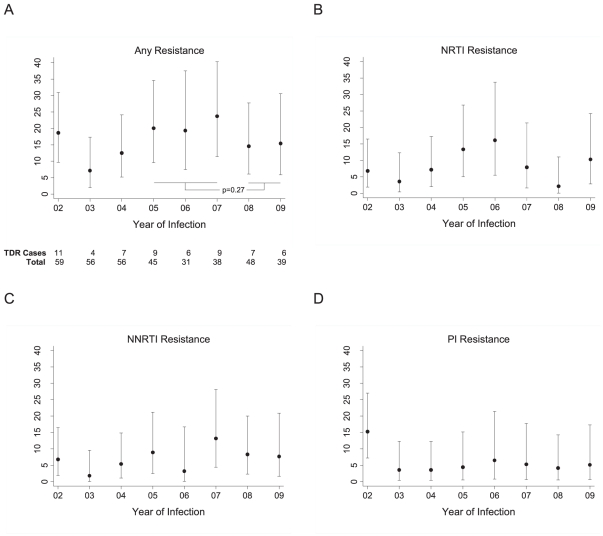
Annual prevalence of transmitted HIV-1 drug resistance in 372 patients with acute/early HIV, 2002-2009. Prevalence (dot) and 95% confidence interval (vertical line) of overall transmitted drug resistance *(A),* NRTI resistance *(B),* NNRTI resistance *(C),* and PI resistance *(D).* TDR, transmitted drug resistance; NRTI, nucleoside reverse transcriptase inhibitor; NNRTI, non-nucleoside reverse transcriptase inhibitor; PI, protease inhibitor.

From 2002–2009, 59 of 372 patients (16%) had transmitted HIV-1 drug resistance mutations. The prevalence of TDR was 19% in 2002, dropped to 7% in 2003, then rose from 2003–2007, reaching a peak of 24% in 2007 (Fig. 1A). Drug resistance was then 15% in 2008, and was also 15% in 2009. NRTI resistance fluctuated substantially, rising from 6% in 2002–2003 to 16% in 2006, then decreasing to 11% in 2009 (Fig. 1B). NNRTI resistance increased from 5% in 2002–2003 to 13% in 2007, and was 8% in 2009 (Fig. 1C). PI resistance was 15% in 2002, but was lower from 2003–2009, ranging from 4–6% (Fig. 1D).

We examined whether TDR differed before and after the arrival of new antiretroviral medications in late 2007. From 2008–2009, 15% (95% CI 9%–25%) of cohort members acquired TDR. This was lower than the 22% prevalence seen in 2005–2007 (95% CI 14%–30%), but the confidence intervals around both point estimates were wide. The odds of TDR was lower in 2008–2009 compared to 2005–2007, but this was not statistically significant (odds ratio 0.65, 95% CI 0.31–1.38; p = 0.27). In unadjusted analyses, age, sexual identity (MSM, male non-MSM, or female), duration of HIV infection, and recent injection drug use all had little association with TDR and did not reach statistical significance, but confidence intervals were too wide to rule out the possibility of substantial associations (data not shown).

## Discussion

We found that despite the introduction of novel ART agents in San Francisco starting in late 2007, including raltegravir, maraviroc, and etravirine, the prevalence of transmitted drug resistance in 2008–2009 remained substantial and was not significantly different than in prior years.

To our knowledge, this is the first report of TDR among persons infected with HIV in 2008–2009, after the newest ART medications arrived. Among patients infected in 2005–2007, Hurt et al. reported a 21% prevalence of TDR in North Carolina, similar to our estimate [1]. Numerous reports exist on TDR trends among persons with chronic HIV, but the impact of new ART is difficult to assess using these data as they can reflect HIV acquisition years before HIV diagnosis.

Our finding that TDR remained substantial in 2008–2009 could have multiple explanations. It is unlikely that new ART medications have inadequate potency given their demonstrated efficacy in treating drug-resistant HIV [7]–[10], [13], [25]. Therefore one explanation for our findings is that suboptimal engagement in medical care and/or poor ART adherence could be limiting the penetration of new ART medications among TDR source patients, leading to a persistently elevated TDR rate. However, recent data from San Francisco investigators demonstrates that virologic suppression is improving, and circulating levels of viremia are decreasing, arguing against poor engagement in care or poor adherence as chief explanations for our results [15].

A second explanation of our findings is that a sizeable fraction of drug-resistant HIV is being transmitted by treatment-naïve persons who themselves acquired TDR. Given that several years typically elapse between HIV infection and diagnosis, ART-naïve individuals have ample time to transmit drug resistance mutations to new recipients. Additionally, persons with early stage HIV are the least likely to be on ART, yet may be responsible for a large fraction of forward transmission events [26]. Furthermore, persons with acute/early HIV may have higher seminal HIV RNA levels, and thus higher infectivity [27]. In this scenario—where TDR is largely transmitted by ART-naïve persons—the effects of novel ART regimens on TDR rates may be delayed.

The TDR prevalence we observed in recent years differs from that predicted by the mathematical model of Smith et al. [16], who had predicted rising NNRTI resistance from 2008–2009. In contrast, our results suggest a stable or even decreasing prevalence of transmitted NNRTI resistance in San Francisco. While the sophisticated aforementioned model provides important insights into TDR patterns, it did not appear to account for changes in HIV treatment options afforded by the newest ART medications, possibly explaining the differences in results.

Our analysis includes several important limitations. First, our acute/early HIV cohort is not a representative population-based sample. As such, our results may not fully reflect the HIV epidemic in San Francisco, though the demographic characteristics of our cohort subjects closely mirror those of the San Francisco epidemic overall. Since our study focused on one geographic area, there are likely to be differences in TDR rates in other settings, particularly in resource-limited regions where treatment options differ substantially. Second, we lack data on the uptake of new ART medications in San Francisco, information which might best be obtained through clinical databases of treatment records but which was not available for this analysis. If the uptake of new ART agents has been slow, more time may be needed to assess the impact on TDR. Third, our results should be interpreted with caution given the wide confidence intervals surrounding the annual estimates for TDR. Studies with longer duration sampling across broader geographic areas may allow better comparisons of TDR before and after the introduction of new ART agents.

Despite these limitations our data provide potential insights into drug-resistant HIV transmission in a setting with historically high levels of drug resistance. Despite the arrival of several novel ART medications beginning in late 2007, the prevalence of TDR remains substantial at the current time. This emphasizes that early diagnosis and aggressive treatment strategies for patients with drug-resistant HIV remain crucial. Continued surveillance will be essential in fully understanding the impact new ART agents will have on TDR epidemiology.
